# Supplementary material for: Sickness absence rates in NHS England staff during the COVID-19 pandemic: Insights from multivariate regression and time series modelling
Source: PLoS One. 2025 Jul 29;20(7):e0323035. doi: 10.1371/journal.pone.0323035 (PMC12306750; doi:10.1371/journal.pone.0323035)
Supplement: Attachment — Submitted filename: Review.pdf [file pone.0323035.s001.pdf]

This research can be recommended for publishing in this journal after revising the MS addressing these issues:

1. I have learned that the author used the SARIMA model. But it is not mentioned what type and how many parameters you considered for the mental health-related absence?
2. This study demonstrated that the mental-related absence is significant than S13, S15, and S27. Is this study able to find the key factors that increase the number of absences for mental health issues? At the same time, what is a suggestion for preventing mental health-related absences?
3. Why is this S10 (mental health-related absence) exponentially increased from March 17, whereas COVID-19 started in December 2019, as shown in Figure 2, panel (a)?
4. What is the limitation of this work and the scope of future study, which is not mentioned here?
